# Snail1 Protein in the Stroma as a New Putative Prognosis Marker for Colon Tumours

**DOI:** 10.1371/journal.pone.0005595

**Published:** 2009-05-18

**Authors:** Clara Francí, Manel Gallén, Francesc Alameda, Teresa Baró, Mar Iglesias, Ismo Virtanen, Antonio García de Herreros

**Affiliations:** 1 Programa de Recerca en Càncer, IMIM-Hospital del Mar, Barcelona, Spain; 2 Servei d'Oncologia, Hospital del Mar, Barcelona, Spain; 3 Servei d'Anatomia Patològica, Hospital del Mar, Barcelona, Spain; 4 Facultat de Medicina, Universitat Autònoma de Barcelona, Barcelona, Spain; 5 Institute of Biomedicine/Anatomy, University of Helsinki, Helsinki, Finland; 6 Departament de Ciències Experimentals i de la Salut, Universitat Pompeu Fabra, Barcelona, Spain; Katholieke Universiteit Leuven, Belgium

## Abstract

Over-expression of Snail1 gene transcriptional repressor promotes an epithelial-to-mesenchymal transition in epithelial tumour cell lines. Expression of Snail1 RNA has been associated to the pathogenesis of a number of malignancies; however, the lack of good monoclonal antibodies against this protein has precluded a definitive analysis of Snail1 protein. In this study, we aimed to determine the expression of this transcriptional factor in colorectal tumours. Using a Snail1 well-characterized monoclonal antibody developed in our laboratories we have analyzed by immunohistochemistry a cohort of 162 human colorectal tumours. Ninety tumours (56%) showed nuclear expression in the tumoral tissue and the adjacent stroma; in 34 (21%), Snail1 was detected just in the stroma, whereas in only 4 the expression of Snail1 was detected in the tumoral tissue and the stroma was negative. No correlation was found between the presence of Snail1 in the tumour and tumour stage; however, a trend (p = 0.054) was detected when the expression of this factor in the stroma was considered. Snail1 immunoreactivity in this compartment was associated with presence of distant metastasis (p = 0.006). Moreover, expression of Snail1 in the tumor stroma correlated with lower specific survival of cancer patients (p = 0.011). Interestingly, this correlation was also detected in stage I and II tumors. Therefore, our results indicate that the presence of nuclear Snail1 immunoreactive cells in the stroma may be an informative indicator of prognosis of colon tumours especially useful in those corresponding to lower stages and identify a new marker suitable to label activated stroma in colon tumours.

## Introduction

Colorectal carcinoma is one of the most common malignancies worldwide [1]. The prognosis of colorectal cancer is fundamentally based on stage. However, some patients eventually die from recurrence and dissemination of cancer soon after surgery, whereas others patients with disease at a similar stage do not. This difference may be the result of the different malignant potential of cancers classified in the same stage. Therefore identification of novel biological markers related to tumour aggressiveness is needed to recognize high risk patients who would benefit from adjuvant therapy and to identify new molecular targets for the development of novel treatments.

Local invasion of carcinomas involves cellular changes associated with a process known as epithelial-mesenchymal transition (EMT), also critical for some early events in embryonic development [2]. The main hallmark of this process is the loss of E-cadherin expression mainly caused by repressed transcription of this gene (CDH1) [2]. Expression of several transcriptional repressors has been shown to down-regulate CDH1 transcription [3]. Among them, an essential role for Snail1 has been highlighted by the general induction of the expression of this gene during EMT in many cell lines and especially by the lack of E-cadherin down-regulation during gastrulation of Snail1 deficient murine embryos [3]. The current working model supposes that Snail1 is required for triggering E-cadherin down-regulation and EMT but not for silencing E-cadherin gene expression in mesenchymal cells [3]. According to this essential role of Snail1 in the modulation of EMT, expression of this factor has been associated to several pathological processes, such as tumour invasion [3] and renal fibrosis [4].

Snail1 expression in adult tissues has been performed by analyzing its corresponding RNA. However, the subcellular localization and stability of this transcriptional factor are sensitive to Ser/Thr phosphorylation [5]–[9] and Lys oxidation of this protein [10]. For instance, GSK-3β-dependent phosphorylation of Snail1 protein translocates this protein to the cytosol, where it is not active, and it is subsequently degraded [5]–[9]. Therefore, Snail mRNA and protein levels do not necessarily correlate. Moreover, Snail1 protein analysis has been hampered by the lack of good antibodies capable to detect this factor in paraffin-embedded samples. We have recently developed a monoclonal antibody (MAb) suitable for this analysis [11]. A preliminary study indicated that Snail1 protein was observed in a small percentage of tumour cells, normally placed at the tumour-stroma interface [11]. Snail1-positive cells were also detected in the stroma [11]. In this article we report the results of a more extensive study performed with 162 colon tumours.

## Results

We have analyzed the expression of Snail1 protein in 162 tumours obtained from colon cancer patients (Table 1). This analysis was carried out using a specific MAb that only detects one band in western blot, reacts with Snail1 protein and not with Snail2 [11], [12]. The specificity of this antibody for the analysis of paraffin-embedded sections was demonstrated by the morphological location of the positive cells detected in embryonic samples and also by the lack of immunoreactivity in sections from Snail1 KO embryos [13]. Expression of Snail1 was detected in 128 of the 162 tumours analysed (79%) and not in the normal tissue obtained from distal areas of the same patients (Table 2). A tumour was considered positive when at least 1% of the cells in the analyzed area showed Snail1 staining. This threshold was chosen in order to compare ours results with previous analysis in other tumours using this cut-off [14]. Only cells with nuclear reactivity were considered to be positive. Cytosolic staining was occasionally detected in our analyses in epithelial cells. This cytosolic reactivity was not considered since, although it may be due to a residual expression of Snail1 protein, this transcriptional factor has been shown to be inactive in the cytosol [5]–[8], rendering its expression outside the nucleus irrelevant.

**Table 1:** Characteristics of 162 patients with colorectal cancer.

Characteristic	N (%)
**Age, mean (±SD)**	68.3 (±11.5)
**Sex**	
Female	67 (41.3%)
Male	95 (58.6%)
**Tumor site**	
Right Colon	43 (26.5%)
Left Colon	85 (52.5%)
Rectum	34 (21%)
**Differentiation of tumor**	
Well	2 (1.2%)
Moderate	152 (93.8%)
Poor	8 (4.9%)
**Histological type**	
Adenocarcinoma (NOS)	143 (88.3%)
Mucinous	19 (11.7%)
**Lymph node metastasis**	
Negative	80 (49.3%)
N1	51 (31.4%)
N2	31 (19.1%)
**Stage**	
I	22 (13.5%)
II	54 (33.3%)
III	65 (40.1%)
IV	21 (12.9%)

**Table 2:** Expression of Snail1 in tumor and stroma according to tumor stage.

		Snail1 expression: tumor (T) or stroma (S)				
		T−/S−	T−/S+	T+/S−	T+/S+	Total
Tumor Stage	I	4	7	1	10	22
	II	13	8	2	31	54
	III	17	13	1	34	65
	IV	0	6	0	15	21
	Total	34	34	4	90	162

Snail1 immunoreactivity was determined in the stromal or carcinoma cells corresponding to colorectal tumours classified in the different stages. According to Snail1 expression, tumours were classified as presenting Snail1 expression both in the tumour and stroma (T+/S+), just in the tumour (T+/S−), just in the stroma (T−/S+) or not present in either of these compartments (T−/S−).

**Figure 1:**
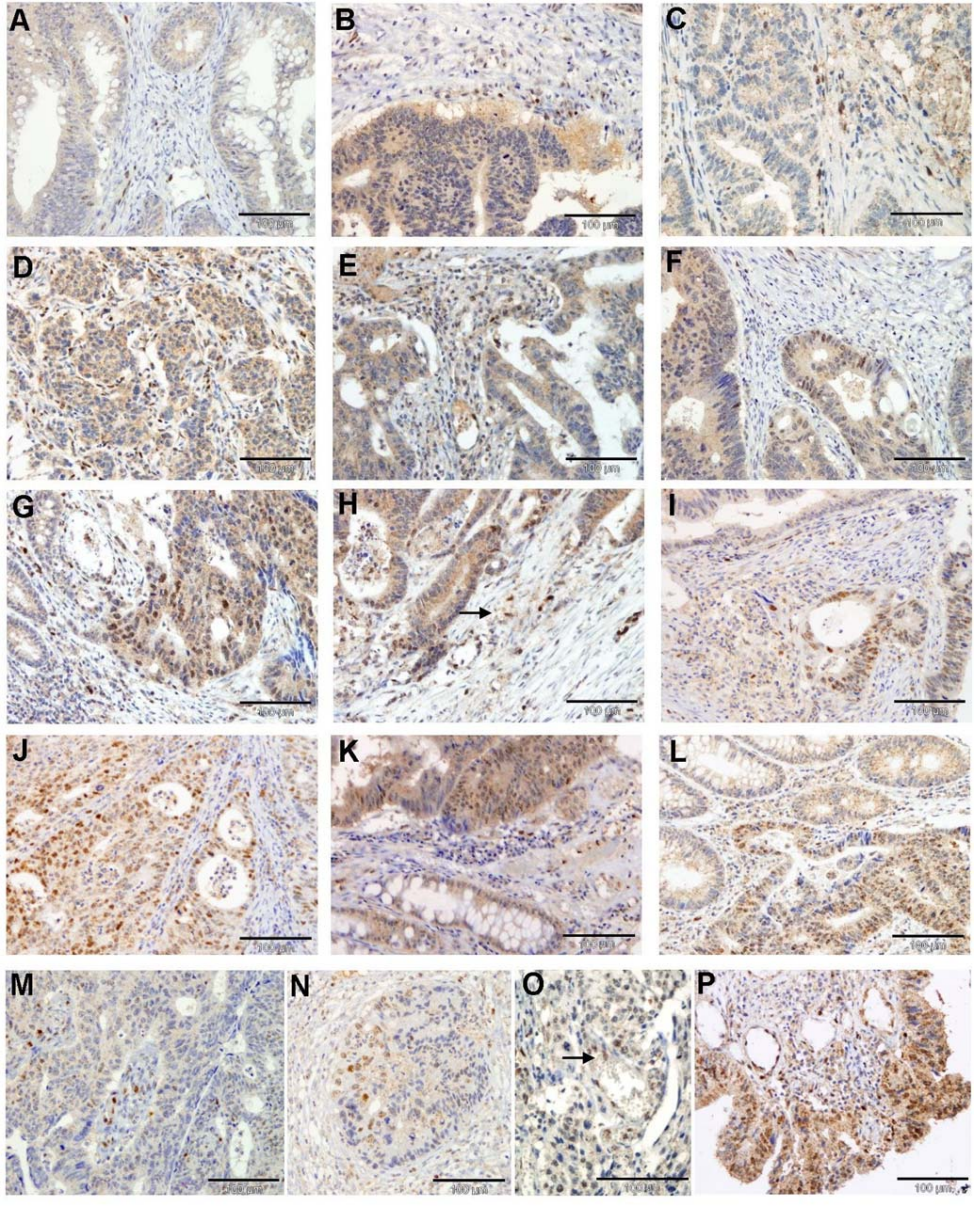
Nuclear Snail1 protein expression in colon carcinomas. Expression of Snail protein was determined as indicated in Methods in samples corresponding to colon carcinomas using MAb EC3. Micrographs of several representative stained sections are shown. Panels A–E corresponded to tumours considered positive only in the stroma; panel F, just in the tumour, and panels G–P; in both compartments. The arrow in panel H labels a cell that cannot be clearly classified as tumoral or stromal. In panel O the arrow points at a cell entering a vessel. Bars indicate magnification.

Representative sections obtained in our analysis are shown in Figure 1. The number of positive cells in the different samples was variable; from tumours with few Snail1 expressing cells (Figure 1A or M) to some cases were Snail1 was massively expressed (Figure 1J). Nuclear immunoreactivity was normally associated with areas of invasion, but not all the invasion fronts were positive. Nuclear expression of Snail1 was more abundant in the stroma. Reactivity in this compartment was observed in spindle fibroblast-like cells and also in histiocytes detected in areas of inflammation. In 34 cases (Figure 1A–E) the immunoreactivity was observed only in the stroma and tumour cells were negative for Snail1 expression. In most cases, positive stromal cells were placed close to the tumoral cells (Figure 1B and C). In 90 samples, positive cells were observed both in the tumour and in the stroma (Figure 1G–P). Frequently, nuclear expression of Snail1 in tumour cells corresponded to areas where the tumour was losing its epithelial structure (Figure 1G, H and I). In some cases, it was not possible to determine if the immunoreactive cell was a carcinoma or stromal cell (for instance, see labelled cell at Figure 1H).

Some other examples of cells presenting Snail1 nuclear expression are also shown. For instance, we detected Snail1 immunoreactive cells migrating out of degenerating glands (Figure 1G, I or N) or in the glandular lumen (Figure 1M). In some occasions Snail1 expressing cells seemed to be entering a vessel, as the labelled cell in Figure 1O. Reactivity in endothelial cells were often detected, as shown in Figures 1O and P. The high expression of Snail1 detected in areas of inflammation (Figure 1P) was also remarkable. Although not common (only 4 cases), the presence of nuclear reactivity in the tumour but not in the stroma was also observed (see Figure 1F). These four samples showed a very low number of positive cells.

We analyzed if Snail1 nuclear expression correlated with clinicopathologic features. Table 2 shows the presence of Snail1 in the tumour and stroma of colorectal tumours at different stages. No significant correlation was found between the expression of Snail1 in the tumour and the tumour stage when we compared columns T+/S− and T+/S+ versus T−/S− and T−/S+ in Table 2. However, when we considered immunoreactivity in the stroma (T−/S+ and T+/S+ versus T+/S− and T−/S−), a trend was obtained with regard to the tumour stage, with a p = 0.053. Since all stage IV tumours presented Snail1-positive cells in the stroma (Table 2), a correlation (p = 0,006) was established between the presence of distant metastasis at the moment of the diagnosis and Snail1 immunoreactivity in the stroma. No significant associations were observed between Snail1 expression in any of the two compartments and other parameters (lymph node metastasis, degree of differentiation or tumour site).

**Figure 2:**
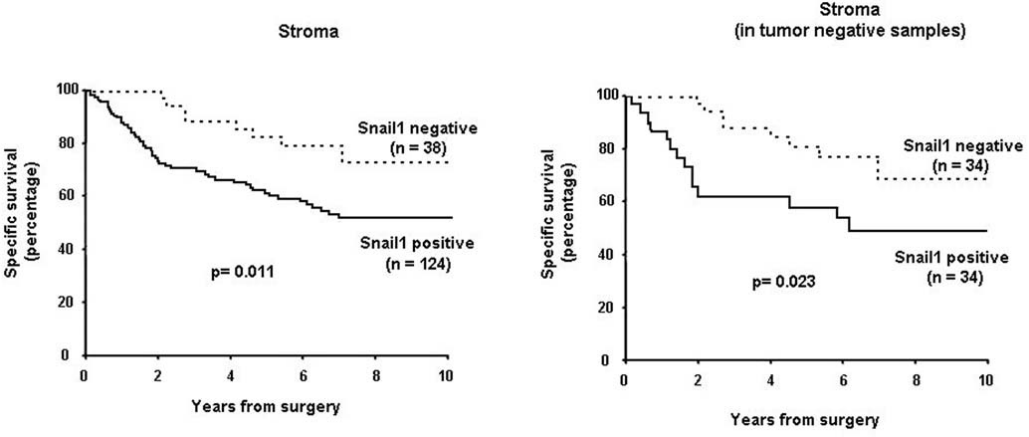
Kaplan-Meier specific survival curves for colon carcinoma patients according to Snail1 expression in the stroma. Discontinuous line represents negative Snail1 immunoreactivity; continuous line, Snail1 positive immunoreactivity. The graphics compare tumours where expression of Snail1 was observed in the stroma with respect to negative ones, regardless of the immunoreactivity in the tumour (left); and with only reactivity in the stroma with respect to negative biopsies (right). The p values are indicated.

The correlation between Snail1 expression and patients' survival was also determined. Specific survival was determined since this parameter reflects the nature of cancer more accurately than overall survival.). As expected according to the higher expression observed in stage IV tumours, presence of Snail1 in the stroma correlated with a lower survival (p = 0.011, see Figure 2, left panel). Additional immunoreactivity in the tumour did not decrease the p value (not shown), giving further indication that the presence of Snail1 in the stroma was the most relevant parameter. Moreover, lower survival was also observed when we compared negative tumours with tumours showing Snail1 reactivity only in the stroma (Figure 2, right panel).

**Figure 3:**
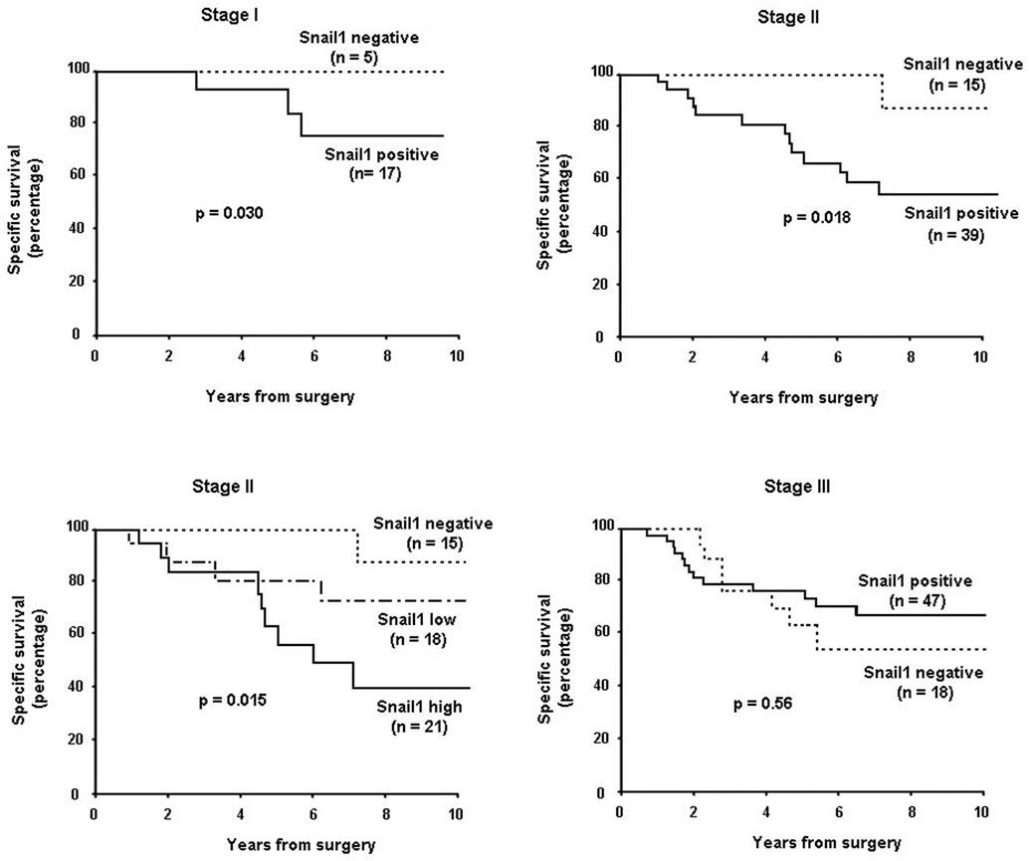
Specific survival of stage I, II and III colon tumour patients according to Snail1 expression in the stroma. The presence of Snail1 in the stroma of stage I, II and III tumours is represented as continuous lines; dotted lines correspond to stroma-negative tumours. In the lower left panel, expression of Snail1 in the stroma was considered as low or high according to the criteria indicated in Methods. The significance is indicated in each category.

We also determined whether Snail1 expression in the stroma of tumours of different stages also correlated with lower survival, regardless its presence in the tumour. This analysis could not be carried out in stage IV tumours since all our specimens were positive for Snail1. We did not find a significant correlation in stage III tumours; however the association between Snail1 immunoreactivity in the stroma and lower survival was significant for stage I and II tumours (Figure 3). Unfortunately, more elaborated statistical analysis could not be performed due to the lack of events in Snail1 negative tumours. However, a Kaplan-Meier analysis was also performed after classifying Snail1-positive samples according to the degree of expression, determined as indicated in Methods. This analysis was only performed on stage II tumours and also demonstrated that presence of Snail1 protein in the stroma correlated with lower survival (Figure 3).

## Discussion

Nowadays it is well accepted that carcinomas must be considered as a complex tissue where signals derived from the stroma play a relevant role in the progression of the disease. Tumour stroma is a complex medium composed of different types of fibroblasts and immune cells recruited by the carcinoma cells. Communication between the tumoral and the stromal compartments has been demonstrated; for example chemokines derived from the tumour can promote the activation of stromal fibroblasts [15]–[17]. Conversely, factors secreted by these cancer activated fibroblasts or other components of the activated stroma have been shown to increase the invasive capability of tumour cells [15], [16] and have been associated to colon tumour progression [18]. Therefore, the determination of specific tumoral markers, not only in the tumoral cells but also in the associated stroma is particularly interesting for determining the progression of this disease.

Using a monoclonal antibody developed in our laboratories, we have analysed the nuclear expression of Snail1 transcriptional factor in human colon tumours. As indicated, this protein works as an E-cadherin-gene repressor, required for triggering EMT. In our analysis Snail1 protein was not detected in normal colonic tissue. However, in a broad percentage of biopsies from colon carcinomas (124/162) it was present in the stroma, or both in the stroma and the tumoral tissue. The percentage of immunoreactive cells in the specimens was variable but expression of Snail1 in positive cases was focalized and detected in areas of invasion or mucosal erosion and ulceration (see Figure 1P). Such a higher expression of Snail1 in areas of ulceration has been detected by other authors using a different MAb [19] and might be due to the response of epithelial cells to cytokines secreted by recruited inflammatory cells.

Analyses of Snail1 gene expression in different types of human tumours have been reported [see 3 for a review]. These studies indicate that Snail1 is associated with invasion, secondary metastasis and poor prognosis [3]. For instance, in breast tumours expression of Snail1 has been associated with relapse [20]. However, most of these studies have been carried out measuring RNA levels and should be interpreted with care since the expression of Snail1 mRNA and the corresponding protein levels do not always correlate, since Snail1 protein is very unstable and its half-life is controlled either by phosphorylation or lysine oxidation (see introduction). Moreover, the cellular localization of Snail1 is also subjected to a post-translational control by phosphorylation [5]. As far as this is concerned, we must emphasize that in our analysis we have only considered as Snail1-positive those cells presenting immunolabelling in the nucleus. The diffuse staining detected occasionally in the cytosol in some epithelial cells was not considered, since Snail1 is not active in this compartment. Another factor that introduces an additional difficulty in the analysis of Snail1 RNA is the existence of a human retrogene with a similar sequence to Snail1 that may interfere with the reverse transcription PCR analysis of Snail1 RNA [21].

The study of Snail1 protein has been hindered by the lack of antibodies suitable for the analysis of paraffin-embedded samples. Several commercial antibodies are available although their specificity has not been fully determined. Anyhow, recent reports have started to analyze Snail1 in human tumours using better characterized antibodies. Becker and co-workers have determined the expression of Snail1 in adenocarcinomas of the upper gastrointestinal tract without detecting any significant association with clinicopathologic parameters [19]. However, these same authors have detected an association of Snail1 expression with tumour grade in endometrioid carcinomas [22] and with overall surviving in ovarian carcinomas [14]. In these studies the association with survival was detected with Snail1 expression in the tumour whereas we found it with its presence in the stroma. It is possible that this different association of Snail1 with clinicopathologic parameters is due to the cell-specific expression of other proteins necessary for the repression by this factor of key targets in epithelial cells, such as E-cadherin. For example, expression of Lysyl oxidase-like 2 protein in Snail1- positive tumour cells may modulate the effects of Snail1 in colon tumour cells, as it has been demonstrated in a recent study with squamous cell carcinomas [23].

In our analysis, the percentage of stage IV tumours showing Snail1 expression in the stroma was significantly higher than for the other stages, suggesting a role for this factor in the promotion of metastasis [3]. In our studies, the most relevant association was detected with the specific survival. Expression of Snail1 in the stroma correlated with lower specific survival in stage I and II tumours that display variable prognosis. The capability to predict recurrence at these stages would have a clinical relevance because markers for this process have not been identified yet. Therefore, the expression of Snail1 would potentially help to identify patients with a worse prognosis and maybe suitable for chemotherapy, a treatment not applied to all early stages colorectal tumours.

As mentioned in the results, the percentage of Snail1 positive cells was low in the tumours. Even considering this low number of reactive cells, we detected a significant correlation with survival, although the tumours presenting higher expression showed lower specific survival. A range of possibilities may explain how Snail1 expression in the stroma, even at lower levels, decreases survival. It is possible that Snail1 is expressed in cells that already have undergone an almost complete EMT and have already evaded from the tumour. Therefore, although Snail1 expression would be limited to the cells that are going through (or have just undergone) EMT, analysis of this factor in the stroma will indicate how many cells have escaped from the tumour and are capable of invading the basal lamina and later on colonize distant target organs. Another alternative would be that higher expression of Snail1 in the stroma may reflect the activation of this compartment. Therefore, a Snail1 immunoreactive stroma would be able to create a richer microenvironment for the progression of colon tumours than a Snail1-negative one. These two possibilities are not mutually exclusive and probably reflect the situation in different areas of the tumour, or at different stages of its evolution. They also agree with the idea that an activated stroma is responsible for generating EMT-induced signals that will favour invasion of tumours cells, as it has been elegantly discussed [24]. In any case they may explain the lower survival of patients showing Snail1 expression in the stroma and support the use of this parameter for the prognosis of stage II colon tumours.

## Materials and Methods

### Patients and tumour samples

We selected 162 patients with colorectal adenocarcinoma who underwent surgery of the primary tumour between January 1995 and December 2001 at the Hospital del Mar, Barcelona. Tumours were obtained from the Tumour Bank from the Servei de Patologia from Hospital del Mar and donated with the written patient's informed consent. The analysis of the samples was approved by the Ethical Committee for Clinical Experimentation of the IMAS (Barcelona, Spain). Clinical data and follow-up were obtained from the review of the patient's medical records and from the Tumour Registry. Postoperative adjuvant chemotherapy with 5-fluorouracil was performed for stage III patients; stage IV patients received palliative chemotherapy. Follow-up of the patients was carried out for at least eight years after surgery. The clinicopathologic characteristics of the patients at the moment of the diagnosis are listed in Table 1. Microscopic confirmation of diagnosis, tumour type and histological grade was carried out by pathologists of Servei de Patologia, Hospital del Mar. Patient staging was classified according to the International Union against Cancer tumour-node metastasis criteria [25]. Specific survival was calculated from time of surgery of the primary tumour to patient death secondary to its colorectal cancer.

### Tissue microarray construction and immunohistochemistry

In order to prepare the tissue microarray, formalin-fixed, paraffin-embedded tissue blocks of colorectal tumours were retrieved from the archives of the Servei de Patologia from Hospital del Mar. Multiple areas of invasive carcinoma and different histological patterns of the tumours (cribiform, mucinous, poorly-differentiated), adenomatous lesions from the same surgical sample, and normal mucosa, located far from the infiltrating tumour, were identified on corresponding haematoxylin-eosin-stained slides. The tissue blocks were transferred to a recipient “master” block using a Tissue Microarrayer. Each core was 0.6-mm wide spaced 0.7–0.8 mm apart.

Immunohistochemical analysis of Snail1 protein was performed as previously described by using MAb EC3 [11]. Tissues were sectioned at 4 µm, deparaffined and rehydrated using xylene and a series of graded ethanol. For antigen unmasking, sections were immersed in Tris EDTA buffer (pH 9), boiled for 20 min, cooled at room temperature for 20 min, and rinsed with PBS. Immunohistochemical staining was carried out with anti-Snail1 MAb EC3 [11] at 1/300 dilution using the CSAII Amplification System (Dako, Glostrup, Denmark), in a Dako Autostainer. Sections were counterstained with haematoxylin.

### Scoring and Statistic al Analysis

Immunohistochemical evaluation was conducted by two investigators who had no knowledge of the clinicopathologic data. Snail1 staining was graded as positive only when nuclear staining was detectable. In each area we separately considered the immunoreactivity of epithelial (tumour) cells and stromal cells (fibroblasts and, in areas of inflammation, histiocytes). Tumour or stroma was considered positive when at least 1% of the cells in the analyzed area showed nuclear staining. A similar threshold for considering a tumour Snail1 positive has been used by other authors [14] when determining the expression of this factor in ovarian tumours. To be considered negative the number of cells in the paraffin block has to be lower than 1%. When analysed tumour microarrays, three sections were analysed in order to avoid false negatives; to be classified as negative the average number of the three sections of the microarray had to show lower than 1% immunoreactive cells. When a semi quantitative evaluation was performed, the staining was scored according to the nuclear staining in a scale of 0 to 300. This was the result of multiplying the percentage of positive cells (from 1 to 100%) and the intensity of immunoreactivity (1 to 3). These samples were categorized into three groups: negative expression; low expression (<10); and high expression (>10).

Survival data were analyzed according to the Kaplan-Meier method and tested for significance between the groups with the log rank test. A p value lower than 0.05 was considered significant. The associations between Snail1 expression and other clinicopathologic variables were assessed by the Chi-square test using categorical variables. All statistical analyses were carried out using StatView for Windows version 5.0 (SAS Institute Inc).

## References

[1] Parker SL, Tong T, Bolden S, Wingo PA (1997). Cancer statistics.. CA Cancer J Clin.

[2] Thiery JP, Sleeman JP (2006). Complex networks orchestrate epithelial-mesenchymal transitions.. Nat Rev Mol Cell Biol.

[3] Peinado H, Olmeda D, Cano A (2007). Snail, Zeb and bHLH factors in tumour progression: an alliance against tumour phenotype?. Nat Rev Cancer.

[4] Boutet A, De Frutos C, Maxwell PH, Mayol MJ, Romero J (2006). Snail activation disrupts tissue homeostasis and induces fibrosis in the adult kidney.. EMBO J.

[5] Domínguez D, Montserrat-Sentís B, Virgós-Soler A, Guaita S, Grueso J (2003). Phosphorylation regulates the subcellular location and activity of the snail transcriptional repressor.. Mol Cell Biol.

[6] Zhou BP, Deng J, Xia W, Xu J, Li YM (2004). Dual regulation of Snail by GSK-3beta-mediated phosphorylation in control of epithelial-mesenchymal transition.. Nat Cell Biol.

[7] Yook JI, Li XY, Ota I, Fearon ER, Weiss SJ (2005). Wnt-dependent regulation of the E-cadherin repressor Snail.. J Biol Chem.

[8] Yook JI, Li XY, Ota I, Hu C, Kim HS (2006). A Wnt-Axin2-GSK3β cascade regulates Snail1 activity in breast cancer cells.. Nat Cell Biol.

[9] Yang Z, Rayala S, Nguyen D, Vadlamudi RK, Chen S (2005). Pak1 phosphorylation of snail, a master regulator of epithelial-to-mesenchyme transition, modulates snail's subcellular localization and functions.. Cancer Res.

[10] Peinado H, Iglesias-de la Cruz MC, Olmeda D, Csiszar K, Fong KS (2005). A molecular role for lysyl oxidase-like 2 enzyme in Snail regulation and tumour progression.. EMBO J.

[11] Franci C, Takkunen M, Dave N, Alameda F, Gómez S (2006). Expression of Snail protein in tumour-stroma interface.. Oncogene.

[12] Takkunen M, Grenman R, Hukkanen M, Korhonen M, García de Herreros A (2006). Snail-dependent and –independent epithelial-mesenchymal transition in oral squamous carcinoma cells.. J Histochem Cytochem.

[13] Escrivà M, Peiró S, Herranz N, Villagrasa P, Dave N (2008). Repression of PTEN phosphatase by Snail1 transcriptional factor during gamma-radiation-induced apoptosis.. Mol Cell Biol.

[14] Blechschmidt K, Sassen S, Schmalfeldt B, Schuster T, Hofler H (2008). The E-cadherin repressor Snail is associated with lower overall survival of ovarian cancer patients.. British J Cancer.

[15] Liotta LA, Kohn EC (2001). The microenviroment of the tumour-host interface.. Nature.

[16] Nelson CM, Bissell MJ (2006). Of extracellular matrix scaffolds and signalling: tissue architecture regulates development, homeostasis, and cancer.. Annu Rev Cell Dev Biol.

[17] Yang J, Weinberg RA (2008). Epithelial-mesenchymal transition: at the crossroads of development and tumour metastasis.. Dev Cell.

[18] Brabletz TA, Jung A, Reu S, Porzner M, Hlubek F (2001). Variable β-catenin expression in colorectal cancers indicates tumour progression driven by the tumour enviroment.. Proc Natl Acad Sci USA.

[19] Rosivatz E, Becker KF, Kremmer E, Schott C, Blechschmidt K (2006). Expression and nuclear localization of Snail, an E-cadherin repressor, in adenocarcinomas of the upper gastrointestinal tract.. Virchows Arch.

[20] Moody SE, Perez D, Pan T, Sarkisian CJ, Portocarrero CP (2005). The transcriptional repressor Snail promotes mammary tumour recurrence.. Cancer Cell.

[21] Locascio A, Vega S, de Frutos CA, Manzanares M, Nieto MA (2002). Biological potential of a functional human SNAIL retrogene.. J Biol Chem.

[22] Blechschmidt K, Kremmer E, Hollweck R, Mylonas I, Hofler H (2007). The E-cadherin repressor Snail plays a role in tumour progression of endometrial carcinomas.. Diagn Mol Pathol.

[23] Peinado H, Moreno-Bueno G, Hardisson D, Pérez-Gómez E, Santos V (2008). Lysysl Oxidase-like 2 as a new prognosis marker of squamous cell carcinomas.. Cancer Res.

[24] Scheel C, Onder T, Karnoub A, Weinberg RA (2007). Adaptation versus selection: the origins of metastatic behaviour.. Cancer Res.

[25] Sobin LH, Wittekind DH International Union Against Cancer (UICC). TNM classification of malignant tumours. 6th, ed.

